# A Real-World Comparison Between Adjuvant Docetaxel with Cyclophosphamide (TC) and Anthracycline–Taxane Chemotherapy in Early HER-2 Negative Breast Cancer

**DOI:** 10.3390/curroncol32010006

**Published:** 2024-12-25

**Authors:** Danilo Giffoni de Mello Morais Mata, Rossanna C. Pezo, Kelvin K. W. Chan, Ines Menjak, Andrea Eisen, Maureen Trudeau

**Affiliations:** 1 Division of Medical Oncology, Verspeeten Family Cancer Centre, London Health Sciences Centre, London, ON N6A 5W9, Canada; 2 Department of Medicine, Schulich School of Medicine and Dentistry, Western University, London, ON N6A 5C1, Canada; 3 ICES Western, London, ON N6A 5W9, Canada; 4 Division of Medical Oncology, Odette Cancer Centre, Sunnybrook Health Sciences Centre, Toronto, ON M4N 3M5, Canada; rossanna.pezo@sunnybrook.ca (R.C.P.); kelvin.chan@sunnybrook.ca (K.K.W.C.); ines.menjak@sunnybrook.ca (I.M.); 5 Department of Medicine, University of Toronto, Toronto, ON M5S 1A1, Canada; 6 Cancer Program, ICES, Toronto, ON M4N 3M5, Canada; 7 Division of Medical Oncology, Juravinski Cancer Centre, Hamilton, ON L8V 1C3, Canada; eisena@hhsc.ca; 8 Hamilton Health Sciences, Hamilton, ON L8V 1C3, Canada

**Keywords:** human epidermal growth factor receptor 2 negative, endocrine (hormone) receptor, paclitaxel, doxorubicin, epirubicin, adjuvant, invasive breast carcinoma

## Abstract

Background: Anthracycline–taxane chemotherapy is the gold standard in high-risk breast cancer (BC), despite the potential risk of congestive heart failure (CHF). A suitable alternative for anthracycline-sparing chemotherapy is through the combination of docetaxel and cyclophosphamide (TC). Methods: Through a retrospective study of stage I-III HER2-negative BC, using administrative databases, we analyzed a total of 10,634 women treated with adjuvant chemotherapy in Ontario, Canada, between 2009 and 2017. We compared TC versus standardized anthracycline–taxane chemotherapies (ACT and FEC-D). We investigated the overall survival (OS), and explored the incidence of CHF, emergency department (ED) visits and febrile neutropenia. Results: With a median follow-up of 5.5 years, the 5-year analysis showed an increased OS in patients treated with TC, versus those treated with ACT, HR 0.77 (0.63–0.95, *p* = 0.015). Among ER+ BC, there was an increased OS in patients treated with ACT and FEC-D, versus those treated with TC, HR 0.70 (0.52–0.95, *p* = 0.021) and HR 0.71 (0.56–0.91, *p* = 0.007), respectively. There were no substantial differences in CHF, between TC and anthracycline-based treatments. Patients treated with TC and FEC-D had more ED visits, compared to those treated with ACT. Conclusion: Our study shows that anthracycline–taxane regimens were the most commonly prescribed adjuvant chemotherapy options in HER2-negative BC. Women who received ACT had the lowest OS, likely due to their unfavorable pathology.

## 1. Introduction

Breast cancer (BC) is the most frequent oncological diagnosis in women, corresponding to approximately 15% of all new cancer diagnoses and 7% of all cancer deaths [1]. A wide array of strategies, such as early tumour detection and better treatment options, have been accessible, improving patients’ life expectancy and leading to a 5-year survival rate of over 90% in non-metastatic breast cancer. Nevertheless, there are more than 50,000 BC-related deaths every year in North America [2]. BC is a heterogeneous disease comprising different morphological subtypes and with varying genomic drivers of signaling pathways, yielding distinct prognoses [3]. Approximately 75% of breast tumours are encoded with luminal differentiation involving endocrine receptor (ER) positivity [4]. Between 15% and 20% of all BC diagnoses feature the overexpression of the human epidermal growth factor receptor 2 (HER2) phenotype, and 10–15% represent triple negative breast cancer (TNBC) [5]. Whereas the luminal BC subtype, ER+ HER2-negative, is typically responsive to endocrine therapy (ET), chemotherapy has an enhanced efficacy with both the HER2+ and basal-like TNBC [6,7,8]. While luminal tumours with a high expression of ER have the most indolent course and most favorable prognosis, the TNBC subtype has the poorest outcomes, likely due to the absence of suitable options for targeted treatments inhibiting specific molecular pathways [4,9,10].

Pathological features associated with a higher likelihood of distant metastasis and worse BC prognosis are linked to increased tumour size, poorly differentiated histology and axillary lymph node involvement (LN+) [11,12]. The latter is strongly correlated with a high-risk of mortality [12]. For example, in ER+ breast cancer, the absolute risk of cumulative breast cancer death at 20 years from the time of diagnosis increases by 13% and 34% in patients with LN+ 1–3 and LN+ ≥ 4, respectively, when compared to patients with unaffected LN [12]. In those lacking ER expression, the TNBC population has the highest rate of distant metastasis and breast cancer mortality, at 5 years [13,14]. Understanding the tumour morphology, e.g., invasive ductal, lobular or mucinous, ER and HER2 expressions, as well as the pathology features and BC stage, are critical to classifying tumours and guiding the clinician’s decision towards the most appropriate treatment for each case [9,15,16].

The role of postoperative systemic therapy is to eliminate latent microscopic malignant cells unidentified in the surgical specimen [9]. When there are clinical or pathological high-risk features and high recurrence risk scores on genomic expression assays, adjuvant chemotherapy is the treatment of choice for improving overall survival (OS) and lowering the risk of local and distant cancer recurrence [17,18,19]. Nonetheless, regardless of BC subtype and which treatment route is taken, relapse events steadily continue beyond the time of chemotherapy completion [20,21]. Conversely, an early treatment start, and adequate adherence, are highly associated with better outcomes [22].

The addition of anthracycline to taxane chemotherapy is the gold standard in the treatment for breast cancer with high risk for cancer relapse. However, the potential short- and long-term side effects have made the choice of anthracycline questionable. Along with the high emetogenic and immunosuppressive characteristics, they can cause irreversible and life-threatening conditions, such as congestive heart failure (CHF) and leukemia [23,24]. Unfortunately, the risk of CHF induced by anthracycline persists after the treatment is completed, leading to an increased absolute risk of 1.72% at 1 year and 3.62% at 10 years post-treatment (*p* < 0.001) [25].

Almost all of the RCTs comparing anthracycline versus non-anthracycline chemotherapy were conducted before the establishment of the 2022 European Society of Cardiology (ESC) guidelines, where it was determined that the evidence of reduced cardiac function is not sufficient to rule out a Cancer Therapy-related Cardiac Dysfunction (CTRCD). Instead, analyzing CHF symptoms is indispensable, since its presentation is often sub-clinical. Thus, it is likely that cardiotoxicity events had been underestimated in many of the RCTs that used anthracycline-based or anti-HER2 therapies, emphasizing the importance of investigating the impact of CHF in retrospective population-based studies [26].

A suitable taxane-based chemotherapy sparing the anthracycline component is the docetaxel and cyclophosphamide (TC) regimen, which has a remarkably safe profile and attains survival outcomes comparable to the anthracyclines in select breast cancer populations [27,28]. For example, in patients who were previously treated with anthracycline or have underlying cardiac predispositions, TC is a reasonable chemotherapy choice [29,30].

Thus, there continues to be debate around the optimal chemotherapeutic option, and in which breast cancer populations may anthracyclines be omitted.

Here, we describe the real-world experience of BC patients treated with adjuvant chemotherapy in Ontario, Canada. We aim to compare the clinical outcomes in patients treated with the TC regimen, versus those receiving the most conventional standardized anthracycline–taxane chemotherapy options.

## 2. Materials and Methods

### 2.1. Study Design

We conducted an observational, retrospective population-based cohort study of adult women diagnosed with stage I-III breast cancer (ICD-10 C50^) between January 2009 and December 2017. We followed the Strengthening the Reporting of Observational Studies in Epidemiology (STROBE) guidelines in the preparation of this manuscript [31].

### 2.2. Patient Population

All patients underwent breast surgery for tumour resection and had a histological diagnosis of invasive breast carcinoma. Patients received adjuvant chemotherapy with TC or anthracycline–taxane-based chemotherapy. Patients were included if their chemotherapy start date was no longer than 120 days from their BC surgery. A minimum of 6 months of follow-up data based on the last registry within the health care databases was required. The end of the follow-up was on 31 March 2018. The exclusion criteria included bilateral BC, previous diagnosis of malignancy, leukemia or CHF, and missing data around demographic information and BC characteristics. Patients were also excluded if they were HER2+ or had an unknown HER2 status but received trastuzumab.

### 2.3. Exposure

Adjuvant chemotherapy was defined as cytotoxic systemic treatments administered intravenously, initiated after the breast surgery was performed. The exposure of interest consisted of the administration of the non-anthracycline TC chemotherapy versus anthracycline–taxane containing regimens. The TC regimen comprised docetaxel and cyclophosphamide administered every 3 weeks for a total of four cycles. The anthracycline–taxane chemotherapy regimens are as follows: fluoracil, epirubicin and cyclophosphamide administered every 3 weeks for 3 cycles, followed by docetaxel every 3 weeks for 3 cycles (FEC-D); doxorubicin and cyclophosphamide were administered every 2 or 3 weeks for a total of four cycles, followed by 12 administrations of paclitaxel, on a weekly schedule, or 4 administrations on a 2-week schedule (ACT). Docetaxel was considered as a substitute for paclitaxel chemotherapy, which was administered every 3 weeks for a total of 4 cycles. Patients treated with paclitaxel on a 3-week schedule were not included because of the inferior outcomes when compared to the standard treatment schedules [32]. The usage of granulocyte-colony stimulating factor (G-CSF) injections was allowed.

### 2.4. Data Sources

Our study data were obtained from ICES (formerly known as the Institute for Clinical Evaluative Sciences); ICES is an independent, non-profit research institute whose legal status, under Ontario’s health information privacy law, allows it to collect and analyze health care and demographic data, without consent, for health system evaluation and improvement. The ICES maintains the health administrative data of more than 14 million Ontario residents [33]. Encrypted identifiers containing anonymous data were used to access patient information and to link them with other health datasets as follows: The Cancer Activity Level Reporting (ALR) and Ontario Cancer Registry (OCR) databases, as well as the registry elements of data of Ontario’s cancer system and the New Drug Funding Program (NDFP), were accessed to verify all Ontario residents diagnosed with BC (ICD-10 C50^) in a patient-level activity focused on postoperative systemic treatment, such as dates, dose, and type of chemotherapy administrations. The Ontario Health Insurance Plan (OHIP), Canadian Institutes of Health Information Discharge Abstract Database (CIHI-DAD), and CIHI National Ambulatory Care Reporting System (CIHI-NACRS) were used to obtain the diagnostic code information of medical procedures, hospital admissions, emergency room visits and discharge diagnosis. It also provided data about the preceding diagnoses of patient comorbidities based on the Charlson–Deyo index. Breast cancer characteristics, including staging, tumour grade, lymph node status, ER and HER2 status, were extracted from the OCR. These datasets were linked using unique encoded identifiers and analyzed at ICES.

Surgical pathology information was used to classify the BC stage. In the case of an unknown HER2 status, patients were classified as HER2 negative if they did not receive trastuzumab. Breast surgery (mastectomy versus lumpectomy and lymph node (LN) surgical assessments (none, sentinel LN biopsy or axillary LN dissection) were identified using procedure codes in the CIHI and Same Day Surgery databases. Radiotherapy was identified from the ALR and OHIP databases. Adjuvant breast radiotherapy was defined as radiation treatment started within 90 days after chemotherapy was completed. The Registered Persons Database (RPDB) provided the demographic information, cause of death and the socioeconomic status, from which the latest median household income quintile by the Canadian postal code were acquired.

This research was approval by the Research Ethics Board of Sunnybrook Health Sciences Centre. Under the legislation of the Ontario Health’s privacy information program, the patients’ written informed consent was waived.

### 2.5. Outcomes

The primary outcome of interest is overall survival (OS). The survival time was defined in months, from the start of adjuvant chemotherapy (index date) to death from any cause. Patients were right-censored at the end of the study follow-up, if no individual death records were encountered.

Secondary outcomes are OS data stratified by the chemotherapy regimens of interest and ER status. A subgroup analysis was used to evaluate OS in ER-positive BC and ER-negative BC, stratified by the chemotherapy regimens, and by the number of axillary LNs. Amongst the chemotherapy options, we compared any diagnosis of congestive heart failure (CHF) from the chemotherapy start date for the rest of patients’ lifetime. We investigated the incidence of febrile neutropenia diagnosis, the most common causes for emergency room visits and hospitalizations, from the chemotherapy starting date to 30 days after its completion. For the respective conditions, we searched for the corresponding International Code Diagnosis (ICD-9 and ICD-10) and acute care visits. To evaluate febrile neutropenia, since there is no specific ICD for this condition, we searched in the administrative database for billing codes associated with acute care visits in women with a diagnosis of early BC, and who were on chemotherapy, postoperatively. For each acute care visit, we used the most responsible diagnosis (MRD). We selected neutropenia as the MRD, plus any diagnosis of fever corresponding to that visit, and created a variable called febrile neutropenia MRD.

### 2.6. Statistical Analysis

We used Kaplan–Meyer methods to analyze the survival curves, based on the adjusted analysis. For the secondary endpoints, we used hazard ratio (HR) estimates in a stratified Cox proportional-hazard regression model (time to event data) for death. For this endeavor, the analysis carried out adjusted the data by all covariates, patient and tumour baseline characteristics, as shown in Table 1. The data were analyzed using the software R (R Foundation for Statistical Computing, Vienna, Austria) version 4.1. All statistical test analyses were two-sided. A *p*-value of ≤0.05 was considered statistically significant. The adjusted analyses were generated. A sensitivity analysis was performed to analyze the potential confounding factor among the independent variables. Multiple imputation was applied to adjust for missing data, using the SAS default single Markov chain Monte Carlo method. Assuming a normal multivariate distribution sample, ten complete datasets were generated for statistical inferences [34].

## 3. Results

### 3.1. Patient Characteristics

**Figure 1:**
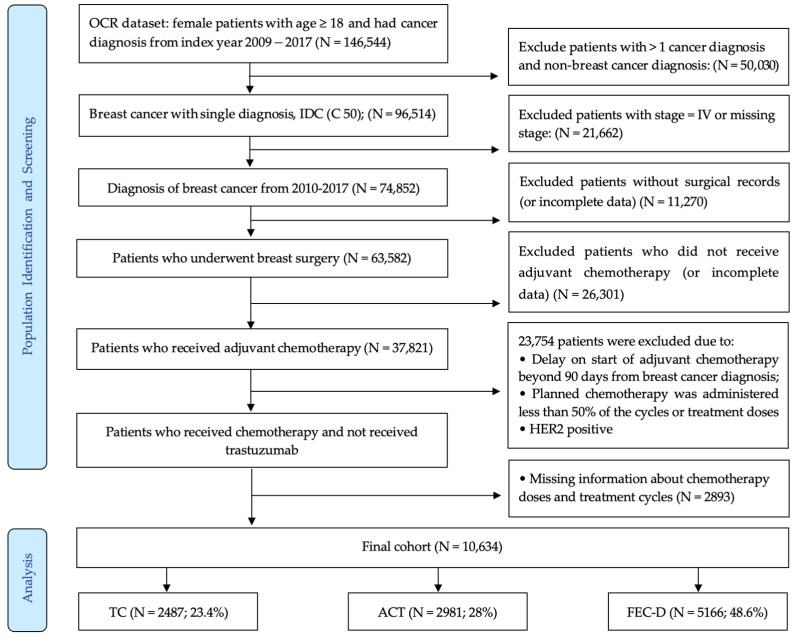
Creation of the cohort of breast cancer patients treated with adjuvant chemotherapy.

We identified a total of 74,852 patients diagnosed with stage I-III BC between 2009 and 2017 in Ontario. From those, there were 37,281 patients treated with adjuvant chemotherapy, plus/minus trastuzumab. A total of 26,633 patients were excluded due to the delayed start of adjuvant chemotherapy beyond 90 days, because they received less than 50% of the planned chemotherapy cycles or treatment doses, or because they had HER2 + BC or an unknown status of HER2, but treated were with trastuzumab. Hence, a total of 10,634 women were included in this study (Figure 1).

The patients’ baseline characteristics are shown in Table 1. A total of 4222 (39.7%) women were between 18 and 50 years old. There were 4970 (46.7%) and 1442 (13.6%) patients between 51 and 65 and older than 65 years of age, respectively.

Approximately 33.3% of patients were similarly treated between each of the following intervals: 2009–2011, 2012–2014 and 2015–2017. The most common underlying comorbidities were hypertension, with 2902 (27.3%) cases reported, followed by diabetes and dyslipidemia, with 1255 (11.9%) and 1016 (9.6%) cases reported. The number of patients on the extreme edges of the income quintile categories, Q1 (lowest) and Q5 (highest), were 1565 (14.7%) and 2564 (24.1%), respectively. A total of 6290 (85.3%) women had an income quintile categorized between Q2–Q4. Patients living in rural areas comprised 1158 (10.9%) of the total number of patients.

Stratifying by BC stage, there were 2087 (19.6%) women with stage I, 6497 (61.1%) with stage II and 2050 (19.3%) with stage III. Among 5764 (54.2%) patients with positive LN, 4300 (40.4%) had LN+ 1–3 and 1464 (13.8%) had LN+ ≥ 4. The number of women with LN 0 was 3832 (36%). There were 3508 (33.0%) patients who had a breast tumour of size 2 cm or smaller, 5216 (49.1%) had tumours between 2 and 5 cm, and 905 (8.5%) had tumours that measured more that 5 cm in size. Regarding the Nottingham histological grading, there were 4945 (46.5%) cases that were considered a grade 3 disease.

A lumpectomy was performed in 6561 (61.7%) patients and a mastectomy was performed in 4073 (38.3%) women. From the final cohort of 10,634 patients, there were 2387 (23.5%) patients treated with TC, 2981 (28.0%) patients who received ACT and 5166 (48.5%) treated with FEC-D chemotherapy regimens. The number of patients who had adjuvant radiation that was started within 90 days from the completion of chemotherapy amounted to a total of 7043 (66.2%).

### 3.2. Overall Survival Analysis

The median follow-up was 5.5 (3.5–7.8) years for the overall study population and 4.6 (3.0–7.2) years for patients treated with TC, 4.5 (2.8–6.5) years for those treated with ACT and 6.7 (4.4–8.6) years for those treated with FEC-D. The median OS of the entire cohort was 8.0 years. After adjusting the analysis by the confounders (age, year of BC diagnosis, Charlson comorbidity index, income quintile, urban/rural residence status, ER, HER2 and LN status, histological grade, type of surgery and radiation treatment), the 5-year OS was 93.8% for those treated with TC, 92.7% for those treated with ACT and 90.7% for those treated with FEC-D, (*p* = 0.052).

**Figure 2:**
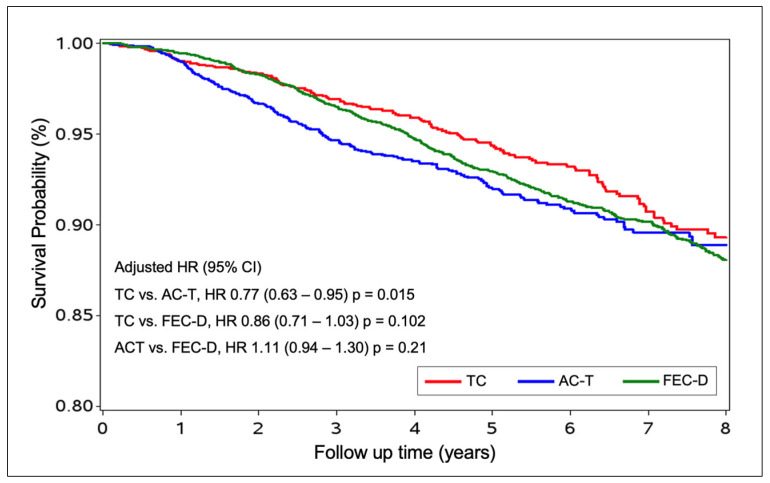
Kaplan–Meyer estimates of the 5-year overall survival adjusted analysis for women with early HER2-negative breast cancer.

The risk of death was higher or not different between those treated with ACT and FEC-D [HR of 1.11 (95% CI, 0.94–1.30), *p* = 0.21], and lower or not different between those treated with TC and FEC-D [HR of 0.86 (95% CI, 0.71–1.03)], *p* = 0.102]. Nevertheless, there was a significantly increased OS, or reduced risk of death, favoring TC vs. ACT [HR of 0.77 (95% CI, 0.63–0.95), *p* = 0.015], (Figure 2).

#### 3.2.1. Overall Survival Analysis by ER Status

**Figure 3:**
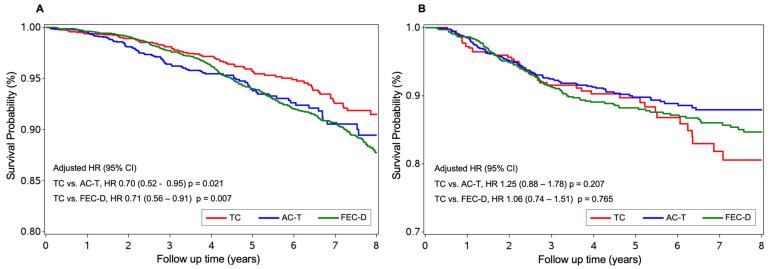
Kaplan–Meyer estimates of the 5-year overall survival adjusted analysis for women with early HER2-negative breast cancer. (**A**): Endocrine receptor positive populations. (**B**): Endocrine receptor negative populations.

A total of 7130 (67.0%) women were ER+ BC, and at the 5-year post-treatment mark, the OS was 95.6% for those treated with TC, 94.4% for those treated with ACT and 92.1% for those treated with FEC-D, *p* = 0.018. The risk of death was significantly lower in patients treated with TC, when compared to those treated with anthracycline–taxane: TC vs. ACT [HR of 0.70 (95% CI, 0.521–0.949), *p* = 0.0218], TC vs. FEC-D [HR of 0.716 (95% CI, 0.56–0.912), *p* = 0.007], (Figure 3A) (Table 2).

A total of 2379 (22.4%) women were ER− BC, and at the 5-year post-treatment mark, the OS was 88.9% for those treated with TC, 91.0% for those treated with ACT and 86.7% for those treated with FEC-D, *p* = 0.321. The risk of death was not different in patients treated with TC chemotherapy, when compared to those treated with the anthracycline–taxane regimens: TC vs. ACT [HR of 1.25 (95% CI, 0.88–1.78), *p* = 0.207]. TC vs. FEC-D [HR of 1.06 (95% CI, 0.74–1.51), *p* = 0.765]. Figure 3B.

#### 3.2.2. Endocrine Receptor Positive

##### Overall Survival Analysis by LN Status

In the adjusted analysis, ER+ BC, patients with LN 0, TC vs. ACT, showed OS HR of 1.17 (95% CI, 0.58–2.35), *p* = 0.67, and TC vs. FEC-D, showed OS HR of 1.38 (95% CI, 0.81–2.33), *p* = 0.23. In patients with LN+ 1–3, TC vs. ACT, OS HR was 1.02 (95% CI, 0.61–1.70), *p* = 0.95, and TC vs. FEC-D, OS HR was 1.17 (95% CI, 0.76–1.80), *p* = 0.49. In patients with LN ≥ 4, TC vs. ACT, OS HR was 1.23 (95% CI, 0.64–2.38), *p* = 0.53, and TC vs. FEC-D, OS HR was 1.27 (95% CI, 0.70–2.32), *p* = 0.43 (Table 3).

Regardless of the chemotherapy regimen received, there was a significant increased death rate, by at least four fold, in patients with LN ≥ 4 when compared to those with LN 0; TC [HR of 4.29 (95% CI, 2.09–8.79) *p* < 0.001]; ACT [HR of 4.13 (95% CI, 2.02–8.45), *p* = 0.001]; FEC-D [HR of 4.94 (95% CI, 3.25–7.50), *p* < 0.001]. In addition, an increased mortality rate was seen in women treated with FEC-D who had LN 1–3 vs. LN 0 [HR of 1.85 (95% CI, 1.22–2.81), *p* = 0.004] (Table 3).

In the multivariate regression model, histological grade 3 diseases [HR of 3.23 (95% CI, 1.34–7.79), *p* = 0.009] and LN ≥ 4, [HR of 4.29 (95% CI, 2.09–8.78), *p* < 0.001] were associated with the increased risk of death in the ER+ group (Table 4).

From the sensitivity analysis, comparing the analyses with versus without multiple imputation for the independent covariates, there was no significant difference in OS in the ER-positive BC population.

#### 3.2.3. Endocrine Receptor Negative

##### Overall Survival Analysis by LN Status

In the adjusted analysis, ER-BC, in patients with LN 0, TC vs. ACT, showed OS HR of 2.04 (95% CI, 1.09–3.81), *p* = 0.025, and TC vs. FEC-D, OS HR of was 2.05 (95% CI, 1.08–3.90), *p* = 0.028. In patients with LN+ 1–3, TC vs. ACT, OS HR was 2.26, (95% CI, 0.84–6.11), *p* = 0.11 and TC vs. FEC-D, OS HR was 1.88 (95% CI, 0.68–5.15) *p* = 0.22. In patients with LN ≥ 4, TC vs. ACT, OS HR was 2.25 (95% CI, 0.79–6.40) *p* = 0.13 and TC vs. FEC-D, OS HR was 2.20 (95% CI, 0.79–6.11) *p* = 0.13 (Table 3).

Similarly to the ER+ group, regardless of the chemotherapy regimen received, a significant increased risk of death, by at least four fold, was observed in those who had LN ≥ 4, compared to those who had LN 0; TC [HR of 4.41 (95% CI, 1.33–14.59), *p* = 0.015]; ACT [HR of 6.70 (95% CI, 3.96–11.35), *p* < 0.001]; FEC-D [HR of 8.48 (95% CI, 4.77–15.05), *p* < 0.001]. In addition, an increased mortality rate was noticed in women with LN 1–3 vs. LN 0, who received ACT [HR of 2.75 (1.71–4.43), *p* < 0.001] and FEC-D [HR of 4.01 (2.43–6.60), *p* < 0.001] (Table 3).

In the multivariate regression model, BC features such as tumour size > 2 to 5 cm [HR of 2.37 (95% CI, 1.11–5.06), *p* = 0.02] and LN ≥ 4 [HR of 4.4 (95% CI, 1.3–14.6), *p* = 0.015)], and the comorbidities CHF [HR of 3.39 (95% CI, 0.98–11.6), *p* = 0.05] and renal disease [HR of 4.26 (95% CI, 1.49–12.2), *p* = 0.006] were all associated with the increased risk of death in the ER– group (Table 4).

From the sensitivity analysis, comparing the analyses with versus without multiple imputation for the independent covariates, there was no significant difference in OS in the ER-negative cohort.

### 3.3. Cardiotoxicity

For this analysis, we evaluated cardiotoxicity when a diagnosis of CHF occurred, from the chemotherapy start day for the rest of the patient’s lifetime. Those who had CHF at baseline (n = 99) were excluded from the analysis (Table 1). A total of 97 patients were diagnosed with CHF during the time this study was conducted. Cardiotoxicity events were found in 25 (1.0%) out of the 2434 patients treated with TC, in 19 (0.6%) out of the 2957 who received ACT, and in 53 (1.0%) out of the 5113 women who were treated with FEC-D. A comparison between TC vs. ACT, revealed a HR of 1.40 (95% CI, 0.77–2.56), *p* = 0.27, and TC vs. FEC-D, showed a HR of 1.28 (95% CI, 0.79–2.55), *p* = 0.32 (Table 2).

### 3.4. Emergency Department Visits and Hospitalization

From 1 January 2009 to 31 December 2017, there were a total of 3600 visits to the emergency department (ED) for BC patients receiving adjuvant chemotherapy. The highest number of ED visits were by those treated with FEC-D (n = 2024; 56.2%), followed by 988 (27.44%) of those treated with TC, and 588 (16.33%) of those treated with ACT, *p* < 0.0001. Through analyzing by chemotherapy received/ED visit, for each 100 breast cancer patients receiving adjuvant chemotherapy, 40 visits to the ED would be expected from those treated with TC, 39 visits from patients treated with FEC-D and 20 visits from the ones who received ACT [RR of 2.0 (95% CI, 1.26–3.16), *p* = 0.003] and [RR of 1.95 (95% CI, 1.22–3.09), *p* = 0.004], respectively. Overall, fever (n = 720; 20%), followed by neutropenia (n = 602; 16.7%) and urinary tract infection (n = 149; 4.14%) were the most frequent diagnosis of those visiting the ED (Table 5).

A total of 1308 hospital admissions were registered during that time frame. The most frequent hospitalizations were for those treated with FEC-D (n = 698; 53.36%), followed by those treated with TC (n = 344; 26.3%) and those treated with ACT (n = 266; 20.3%), *p* < 0.0001. For every 100 breast cancer patients receiving adjuvant chemotherapy, 14 hospitalizations would be expected from those treated with FEC-D, 14 from patients treated with ACT, and 9 from the ones treated with TC [RR of 0.61 (95% CI, 0.25–1.48) *p* = 0.27]. Overall, neutropenia (n = 558; 45%), fever (n = 95; 7.26%) and pneumonia (n = 33; 2.52%) were the most frequent hospitalization diagnosis (Table 5).

## 4. Discussion

Through a retrospective cohort design, we conducted a population-based real-world study using the administrative data of 10,634 breast cancer patients treated in Ontario, Canada. All patients received chemotherapy after having undergone BC surgery for tumour resection. We compared the treatment efficacy between the TC, ACT and FEC-D chemotherapy regimens.

With a median follow-up of 5.5 years, the analysis of the entire population showed a significantly shorter OS in patients treated with ACT compared to those treated with TC [HR of 0.77 (95% CI, 0.63–0.95), *p* = 0.015]. No substantial differences were seen in OS between those treated with ACT vs. FEC-D.

Among patients with ER+ BC, there was a reduced OS in patients treated with ACT or FEC-D, compared to those treated with TC. In the analysis comparing TC vs. ACT or FEC-D treatments, no OS differences were found in the subgroup of patients with LN0, LN 1–3 and LN ≥ 4.

In the ER-BC group, no significant survival differences were seen between the TC and any of the anthracycline-based treatments. Although TC was associated with an increased mortality risk in all LN groups, when compared to ACT and FEC-D, a statistically significant reduction in OS was only observed in those treated with TC who had LN 0. The reason for this is not clear. In the ER-BC population, amid the 2379 women and 247 events of deaths registered in that group, there were 1626 (68.3%) patients and 88 (5.4%) deaths in the LN 0 cohort. In the LN 1–3 group, there were 85 (15.6%) deaths among the 542 patients, and in those with LN ≥ 4, there were 74 (37%) deaths among the total of 200 patients. Three patients had missing data in the ER-BC cohort. These findings lead to the assumption that the sample size of the studied population was less likely statistically powered for a time-to-event analysis in the LN 0, and more likely statistically powered in the LN 1–3 and LN ≥ 4 groups.

The predicting risk factors associated with the poorest outcomes in ER+ were a histological grade of 3 and LN ≥ 4, and those in the ER− group were tumour size (≥2–5 cm), LN ≥ 4, and underlying CHF and renal disease.

Exploring the potential reasons for the finding of an increased risk of death related to anthracycline–taxane treatments, we found that this could be associated with their use in a large majority of patients with high risk features for BC recurrence, including a younger age, LN+, and tumours larger than 2 cm. Among all the treatments compared, the higher mortality associated with ACT could be associated with the group possessing a more unfavorable BC morphology, as 60.2% of patients had a histological grade of 3 and 41.8% had TNBC. In summary, the population selected to receive anthracycline–taxane treatments were mostly at a higher risk for relapse or had a high burden of disease.

Regarding CHF diagnosis, our study did not find significant cardiotoxicity events in the comparison between TC and any of the anthracycline–taxane regimens.

In our study, a total of 48.6% of patients were treated with FEC-D chemotherapy. Thus, this group had significantly more ED visits (56.22%) and hospitalizations (53.36%), compared to those treated with TC or ACT. However, these absolute numbers do not reflect the real proportion when analyzed by treatment received/event (ED visit or hospital admission). Thus, using this parameter, patients who were treated with ACT had significantly less ED visits, when compared to TC and FEC-D. No substantial relative risk difference was identified between the last two chemotherapy comparisons. There was no statistically significant increased risk of hospitalization between any of the chemotherapy options.

As the main causes for ER visits and hospitalizations were fever/neutropenia, and the specific proportion of patients who were given G-CSF prophylaxis in our study is undetermined, it is hypothesized that the absence of prophylaxis with G-CSF in women receiving FEC-D treatment could, to some extent, justify those high rates. During the timeframe of this study, the G-CSF was covered for the dose-dense ACT regimen. However, only after 2012 did the G-CSF turned out to be publicly funded, at a provincial level, for all chemotherapy regimens that could lead to at least 20% of febrile neutropenia events, and this included the FEC-D regimen.

### 4.1. Comparison with Other Studies

Our study showed similar results that have been demonstrated by the combined analysis of PLANB and SUCCESS C trials, which recruited 5924 patients with HER2-negative BC with pathological intermediate and high-risk factors for recurrence. Regardless of the breast cancer ER status, these studies did not show a 5-year OS benefit for adjuvant anthracycline–taxane over TC chemotherapy, with a HR of 1.00 (95% CI, 0.79–1.25, *p* = 0.99) [35]. A recent joint efficacy analysis by the Anthracyclines in Early Breast Cancer (ABC) trials included 4242 women with HER2-negative BC, from the USOR 06-090, NSABP B-46-I/USOR 07132, and NSABP B-49 controlled trials. These study results did not demonstrate any OS difference between TC and anthracycline–taxane chemotherapy, with a HR of 1.05 (95% CI, 0.87–1.26; *p* = 0.64) at 5-year [27].

A recently systematic-review and meta-analysis included seven RCTs and 11,803 women with early HER2-negative BC. This study showed a high quality of evidence that there is no OS advantage between TC vs. anthracycline–taxane, with a HR of 1.02 (95% CI, 0.89–1.16, *p* = 0.83) [36].

An observational study of women with ER+ HER2-negative breast cancer, with a high-risk score on MammaPrint^®^, aimed to predict the anthracycline sensitivity, by analyzing the pathologic complete response (pCR), after neoadjuvant chemotherapy. Patients were treated with TC or anthracycline–taxane. In patients classified into the MammaPrint^®^ High-1 risk category, a similar chemotherapy effectiveness was seen between the two chemotherapy regimens, revealing no difference in pCR rates. Nevertheless, in the group where the risk of recurrences was higher, the High-2 category, anthracycline–taxane was substantially more efficacious than TC (pCR rate 32% vs. 0%, *p* < 0.001). Through the lens of genomic expression assays (GEAs), it is unknown whether the chemotherapy efficacy outcomes could translate into OS and DFS differences between TC and anthracycline–taxane, in neoadjuvant or adjuvant settings, in patients with high-risk scores on GEAs [37].

It is imperative to underscore that there have not been any RCTs comparing the effectiveness between TC chemotherapy, delivered in four cycles (TCx4) versus six cycles (TCx6), or the comparison between TCx4 (rather than TCx6) versus anthracycline–taxane chemotherapy. Another retrospective real-world cohort study has examined the comparison between TCx4 and TCx6, finding no disparity in the relapse and mortality rates between the two regimens, and noting fewer treatment toxicities among those treated with TCx4 [38]. The US Oncology 9735 randomized trial also showed that TCx4 was more effective than the combination of anthracycline and cyclophosphamide (AC), yielding improvements in DFS and OS [39]. As a result, many oncology guidelines recommend the use of TCx4 for adjuvant BC treatment [40].

In our study, in the ACT group, we included patients treated with paclitaxel delivered on a dose-dense schedule and paclitaxel administered weekly, since both are comparable treatment strategies [41].

Regarding cardiotoxicity events, our cohort study showed similar results, compared to a recent systematic review and meta-analysis of RCTs. Their study analyzed 9732 women with HER2-negative BC, and they found high-quality evidence to indicate that there is no significant difference in CHF diagnosis between patients treated with adjuvant TC vs. anthracycline–taxane chemotherapy [36].

Previous reports indicated that 6% of the patients who had been administered with anthracyclines demonstrate clinically evident cardiotoxicity, with as many as 18% developing subclinical congestive heart failure [42]. The factors that increase the risk of developing cardiotoxicity include the cumulative dose of anthracycline, radiation therapy to the left chest, African American ethnicity, diabetes, hypertension, and severe underlying health conditions. The addition of trastuzumab plus/minus pertuzumab in HER2 + BC significantly raises the likelihood of cardiac dysfunction in patients previously exposed to anthracyclines [25,42]. Numerous studies examining anthracycline-induced CHF included cancer patients who received monoclonal antibodies targeting the HER2 domain, potentially leading to an overestimation of anthracycline’s cardiotoxicity risk [25,26].

An observational study of cancer patients treated with chemotherapy in Ontario, Canada, showed that the most frequent ED visits were related to fever or infection [43]. Two retrospective cohort studies of women with early breast cancer treated with adjuvant chemotherapy, in Canada, evaluated similar endpoints. Pittman et al. showed that the most frequent cause of ED visits were fever without neutropenia (23.3%), pain (12.8%), and febrile neutropenia (9%) [44]. In the study by Torres et al., the most common ED visit and hospitalization diagnosis were fever and neutropenia, respectively [45]. Moreover, in the latter study, there was a significant increase in acute care visits and hospital admission in patients who received FEC-D chemotherapy [45].

### 4.2. Strengths and Limitations

To our knowledge, there has not yet been a published paper about the BC population in Ontario, Canada, comparing the survival-related endpoints between TC and anthracycline–taxane based chemotherapy in HER2-negative BC treated in adjuvant settings.

Through a descriptive and observational cohort study, we conducted a comprehensive real-world evidence-based analysis of women with early HER2-negative BC treatedwith TC versus anthracycline–taxane chemotherapy. We utilized solid and reliable individual-level administrative data of individuals registered in our Health Care System in Ontario, Canada. To achieve this endeavor, we succinctly refined the study population by excluding those with missing data of treatment doses and schedule, patients’ baseline characteristics and BC surgical information. We aimed to maximize the accuracy of chemotherapy efficacy, and we excluded patients who received less than 50% of the planned chemotherapy administration doses and schedule. Thus, these attempts reduced the eligible population.

To reduce confounding and selection bias, the final analysis was adjusted by relevant patients’ prognostic and BC characteristics, and we conducted a sensitivity analysis and multiple imputation.

Patients with an advanced age are commonly underrepresented in RCTs, with a potentially inaccurate replication of a genuine clinical environment of BC patients due to the clinical trial setting. Our retrospective cohort study had a satisfactory number of patients who were 65 years or older.

Our study carried out a reasonably prolonged median follow-up duration of 5.5 years, satisfactory to observe death events to accomplish the study endpoints.

The administrative health database utilized for this work is susceptible to limitations. For example, there is a risk of the misclassification of the diagnostic code used to evaluate the cause of ED visits, hospitalizations, and febrile neutropenia diagnosis. Our database is also susceptible to underreporting. For example, women’s menopausal status, compliance with endocrine therapy, and chemotherapy adverse events were not reported. As a result, we could not investigate the usage of endocrine therapy in patients with ER+ BC, or to compare the safety profile between treatments.

With regards to the use of propensity score matching (PSM) to balance patients’ baseline characteristics, there were limitations about this method that could be applicable to our study. From our perspective, implementing a PSM might have actually led to an increased imbalance, rather than a reduction, particularly in terms of increasing post-matching covariate imbalance. Therefore, we decided against using PSM.

Prior to the era of genomic medicine, the clinician’s decision about which ER+ HER − BC patient would benefit from receiving adjuvant chemotherapy was based on increased risk features from the surgical specimen. GEAs, such as OncotypeDX^®^ or MammaPrint^®^, indicate that, many times, chemotherapy is not advantageous for patients with a high risk of cancer relapse, and overtreatment should be avoided [18,19]. It is important to highlight that, according to the time frame of this study from which patients were treated with chemotherapy, it is likely that most of the ER+ HER2-negative patients did not have GEA, and perhaps not all patients included in this study would have derived a substantial benefit from chemotherapy. Moreover, at the time this study was conducted, the advantage of immune checkpoint inhibitors on the treatment of TNBC was unknown. To date, the addition of pembrolizumab to chemotherapy, preoperatively, has shown to be efficacious in improving the rates of pCR, and in reducing BC relapse events, when added to the adjuvant setting [46,47].

## 5. Conclusions

Our study shows that anthracycline-containing regimens were the most largely prescribed adjuvant chemotherapy option for women with early HER2-negative BC. Among the treatment comparisons, ACT had the lowest OS. These patients were mostly at a higher risk for relapse, as they were predominantly young, and had large and poorly differentiated tumours. Similarly, women who had ER+ BC, and received ACT or FEC-D, had the poorest OS, when compared to TC, likely due to their unfavorable BC features.

## Figures and Tables

**Table 1:** Patients’ baseline characteristics.

Variable	Total	TC	ACT	FEC-D	*p*
	(N = 10,634)	(N = 2487)	(N = 2981)	(N = 5166)	Value
Index year group					
2009–2011	3526 (33.2%)	635 (25.5%)	540 (18.1%)	2351 (45.5%)	<0.0001
2012–2014	3541 (33.3%)	802 (32.2%)	1063 (35.7%)	1676 (32.4%)	
2015–2017	3567 (33.5%)	1050 (42.2%)	1378 (46.2%)	1139 (22.0%)	
Age Group					
18–50	4222 (39.7%)	643 (25.9%)	1309 (43.9%)	2270 (43.9%)	<0.0001
51–65	4970 (46.7%)	1160 (46.6%)	1369 (45.9%)	2441 (47.3%)	
>65	1442 (13.6%)	684 (27.5%)	303 (10.2%)	455 (8.8%)	
Surgery type					
Lumpectomy	6561 (61.7%)	1733 (69.7%)	1791 (60.1%)	3037 (58.8%)	<0.0001
Mastectomy	4073 (38.3%)	754 (30.3%)	1190 (39.9%)	2129 (41.2%)	
Breast cancer stage					
I	2087 (19.6%)	979 (39.4%)	482 (16.2%)	626 (12.1%)	<0.0001
II	6497 (61.1%)	1356 (54.5%)	1906 (63.9%)	3235 (62.6%)	
III	2050 (19.3%)	152 (6.1%)	593 (19.9%)	1305 (25.3%)	
Histological grade					
1	743 (7%)	241 (9.7%)	148 (5.0%)	354 (6.9%)	<0.0001
2	3800 (35.7%)	1079 (43.4%)	850 (28.5%)	1871 (36.2%)	
3	4945 (46.5%)	995 (40.0%)	1794 (60.2%)	2156 (41.7%)	
Unknown	1146 (10.8%)	172 (6.9%)	189 (6.3%)	785 (15.2%)	
Tumour size					
≤2 cm	3508 (33.0%)	1248 (50.2%)	876 (29.4%)	1384 (26.8%)	<0.0001
>2–5 cm	5216 (49.1%)	994 (40.0%)	1681 (56.4%)	2541 (49.2%)	
>5 cm	905 (8.5%)	105 (4.2%)	277 (9.3%)	523 (10.1%)	
Unknown	1005 (9.4%)	140 (5.6%)	147 (4.9%)	718 (13.9%)	
Number of lymph nodes					
0	3832 (36%)	1434 (57.7%)	1145 (38.4%)	1253 (24.3%)	<0.0001
1–3	4300 (40.4%)	807 (32.4%)	1227 (41.2%)	2266 (43.9%)	
≥4	1464 (13.8%)	96 (3.9%)	453 (15.2%)	915 (17.7%)	
Unknown	1038 (9.8%)	150 (6.0%)	156 (5.2%)	732 (14.2%)	
ER status					
Negative	2379 (22.4%)	397 (16.0%)	1246 (41.8%)	736 (14.2%)	<0.0001
Positive	7130 (67%)	1924 (77.4%)	1546 (51.9%)	3660 (70.8%)	
Unknown	1125 (10.6%)	166 (6.7%)	189 (6.3%)	770 (14.9%)	
HER2 status					
Negative	6824 (64.2%)	1790 (72.0%)	2336 (78.4%)	2698 (52.2%)	<0.0001
Unknown *	3810 (35.8%)	697 (28.0%)	645 (21.6%)	2468 (47.8%)	
Comorbidities					
CHF	99 (0.9%)	53 (2.1%)	24 (0.8%)	22 (0.4%)	<0.0002
HTN	2902 (27.3%)	833 (33.5%)	697 (23.4%)	1372 (26.6%)	<0.0001
Diabetes	1266 (11.9%)	387 (15.6%)	293 (9.8%)	586 (11.3%)	<0.0001
CKD	161 (1.5%)	55 (2.2%)	51 (1.7%)	55 (1.1%)	0.0004
Charlson Comorbidity Index					
Mean ± S.D.	2.75 ± 2.61	2.22 ± 2.42	2.57 ± 2.54	3.07 ± 2.68	<0.0001
0	2092 (19.7%)	508 (20.4%)	626 (21.0%)	958 (18.5%)	<0.0001
1–2	2260 (21.2%)	534 (21.5%)	630 (21.1%)	1096 (21.2%)	
3–4	355 (3.3%)	121 (4.9%)	84 (2.8%)	150 (2.9%)	
≥5	2103 (19.8%)	279 (11.2%)	529 (17.8%)	1295 (25.1%)	
Unknown	3824 (36.0%)	1045 (42.0%)	1112 (37.3%)	1667 (32.3%)	
Income Quintile					
(Lowest) Q1	1565 (14.7%)	325 (13.1%)	415 (13.9%)	825 (16.0%)	<0.0001
Q2	1981 (18.7%)	451 (18.2%)	579 (19.5%)	951 (18.4%)	
Q3	2146 (20.2%)	516 (20.8%)	587 (19.7%)	1043 (20.2%)	
Q4	2363 (22.3%)	517 (20.8%)	654 (22.0%)	1192 (23.1%)	
(Highest) Q5	2564 (24.1%)	673 (27.1%)	741 (24.9%)	1150 (22.3%)	
Rural	1158 (10.9%)	292 (11.7%)	383 (12.9%)	483 (9.3%)	<0.0001

ER: endocrine receptor; CHF: congestive heart failure; HTN: hypertension; CKD: chronic kidney disease; S.D. standard deviation. * Unknown/missing HER2 status: patients who were not HER2 positive or negative and did not receive trastuzumab.

**Table 2:** Adjusted analysis of overall survival and congestive heart failure in early HER2-negative breast cancer.

Overall Survival	Adjusted Analysis		Congestive Heart Failure	Adjusted Analysis	
Chemotherapy Comparison	HR (95% CI)	*p* Value	Chemotherapy Comparison	HR (95% CI)	*p* Value
TC vs. ACT	1.29 (1.05–1.59)	*p* = 0.015	TC vs. ACT	0.71 (0.39–1.29)	*p* = 0.270
TC vs. FEC-D	1.16 (0.97–1.40)	*p* = 0.102	TC vs. FEC-D	0.78 (0.39–1.26)	*p* = 0.320

**Table 3:** Adjusted analysis of overall survival in ER-positive and ER-negative patients by lymph node status and chemotherapy treatment.

Overall Survival ER+		Adjusted Analysis		Overall Survival ER−		Adjusted Analysis	
	Chemotherapy Comparison	HR (95% CI)	*p* Value		Chemotherapy Comparison	HR (95% CI)	*p* Value
	TC vs. ACT	1.42 (1.05–1.91)	*p* = 0.022		TC vs. ACT	0.80 (0.56–1.13)	*p* = 0.207
	TC vs. FEC-D	1.39 (1.01–1.78)	*p* = 0.007		TC vs. FEC-D	0.94 (0.66–1.35)	*p* = 0.765
LN Status				LN Status			
LN 0	TC vs. ACT	1.17 (0.58–2.35)	*p* = 0.670	LN 0	TC vs. ACT	2.04 (1.09–3.81)	*p* = 0.025
	TC vs. FEC-D	1.38 (0.81–2.33)	*p* = 0.230		TC vs. FEC-D	2.05 (1.08–3.90)	*p* = 0.028
LN 1–3	TC vs. ACT	1.02 (0.61–1.70)	*p* = 0.950	LN 1–3	TC vs. ACT	2.26 (0.84–6.11)	*p* = 0.110
	TC vs. FEC-D	1.17 (0.76–1.80)	*p* = 0.495		TC vs. FEC-D	1.88 (0.68–5.15)	*p* = 0.220
LN ≥ 4	TC vs. ACT	1.23 (0.64–2.38)	*p* = 0.531	LN ≥ 4	TC vs. ACT	2.25 (0.79–6.40)	*p* = 0.132
	TC vs. FEC-D	1.27 (0.70–2.32)	*p* = 0.430		TC vs. FEC-D	2.20 (0.79–6.11)	*p* = 0.131
Chemotherapy	LN Comparison			Chemotherapy	LN Comparison		
TC	LN 1–3 vs. LN 0	1.34 (0.81–2.21)	*p* = 0.262	TC	LN 1–3 vs. LN 0	1.12 (0.42–3.01)	*p* = 0.820
	LN ≥ 4 vs. LN 0	4.29 (2.09–8.79)	*p* < 0.001		LN ≥ 4 vs. LN 0	4.41 (1.33–14.59)	*p* = 0.015
ACT	LN 1–3 vs. LN 0	1.74 (0.86–3.35)	*p* = 0.130	ACT	LN 1–3 vs. LN 0	2.75 (1.71–4.43)	*p* < 0.001
	LN ≥ 4 vs. LN 0	4.13 (2.02–8.45)	*p* = 0.001		LN ≥ 4 vs. LN 0	6.70 (3.96–11.35)	*p* < 0.001
FEC-D	LN 1–3 vs. LN 0	1.85 (1.22–2.81)	*p* = 0.004	FEC-D	LN 1–3 vs. LN 0	4.01 (2.43–6.60)	*p* < 0.001
	LN ≥ 4 vs. LN 0	4.94 (3.25–7.50)	*p* < 0.001		LN ≥ 4 vs. LN 0	8.48 (4.77–15.05)	*p* < 0.001

ER: endocrine receptor; LN: lymph node.

**Table 4:** Multivariate cox proportional regression model for death in the ER-positive and ER-negative breast cancer populations.

Variable	ER+ Cohort					ER− Cohort				
	P.E.	S.E.	Chi^2^	HR (95% CI)	*p* Value	P.E.	S.E.	Chi^2^	HR (95% CI)	*p* Value
Index year										
2012–2014	0.4	0.29	1.9	1.49 (0.84–2.65)	0.16	−0.26	0.43	0.4	0.76 (0.32–1.79)	0.53
2015–2017	0.62	0.37	2.8	1.87 (0.90–3.89)	0.09	−0.36	0.50	0.5	0.69 (0.25–1.87)	0.46
Age group										
51–65	0.33	0.31	1.1	1.39 (0.75–2.58)	0.29	1.75	1.07	2.6	5.75 (0.69–47.5)	0.1
>65	0.57	0.35	2.6	1.78 (0.88–3.60)	0.10	1.43	1.08	1.8	4.20 (0.50–35.2)	0.18
Type of surgery										
Mastectomy	0.31	0.23	1.8	1.37 (0.86–2.18)	0.18	0.81	0.38	4.6	2.25 (1.06–4.73)	0.03
Histological grade										
II	0.38	0.45	0.7	1.46 (0.60–3.54)	0.39	14.2	1016	0	133.1 (0.00–NR)	0.98
III	1.17	0.45	6.8	3.23 (1.34–7.79)	0.009	14.0	1016	0	129.1 (0.00–NR)	0.98
Tumour size										
>2–5 cm	0.33	0.24	1.9	1.40 (0.87–2.24)	0.16	0.87	0.38	5	2.37 (1.11–5.06)	0.02
>5 cm	0.29	0.49	0.3	1.34 (0.50–3.53)	0.55	0.58	0.86	0.5	1.79 (0.33–9.72)	0.49
Number of lymph nodes										
1–3	0.29	0.25	1.3	1.33 (0.81–2.21)	0.25	0.11	0.50	0	1.11 (0.41–3.0)	0.82
≥4	1.45	0.36	16	4.29 (2.09–8.78)	< 0.001	1.48	0.61	5.9	4.40 (1.3–14.6)	0.01
Comorbidities										
CHF	−0.42	0.75	0.3	0.65 (0.14–2.88)	0.57	1.22	0.63	3.8	3.39 (0.98–11.6)	0.05
HTN	0.02	0.24	0	1.02 (0.63–1.66)	0.92	−0.078	0.38	0	0.92 (0.43–1.95)	0.83
Diabetes	0.42	0.27	2.4	1.53 (0.88–2.64)	0.12	−0.004	0.41	< 0.01	0.99 (0.44–2.24)	0.99
CKD	0.22	0.73	0.1	1.24 (0.29–5.30)	0.76	1.45	0.53	7.3	4.26 (1.49–12.2)	0.006
Income quintile										
Q2	−0.1	0.37	0.1	0.90 (0.43–1.87)	0.78	0.36	0.56	0.4	1.44 (0.47–4.36)	0.51
Q3	−0.36	0.38	0.9	0.69 (0.32–1.46)	0.33	−0.10	0.59	0	0.89 (0.28–2.86)	0.85
Q4	−0.27	0.37	0.5	0.76 (0.36–1.57)	0.46	0.41	0.53	0.6	1.51 (0.53–4.30)	0.43
Q5	−0.19	0.35	0.3	0.82 (0.41–1.63)	0.59	−0.33	0.59	0.3	0.71 (0.22–2.29)	0.57
Rural	−0.66	0.41	2.6	0.51 (0.22–1.15)	0.10	0.01	0.49	0	1.01 (0.38–2.69)	0.97

P.E.: parameter estimate; S.E.: standard error; ER: endocrine receptor; HR: hazard ratio; CHF: congestive heart failure; HTN: hypertension; CKD: chronic kidney disease; NR: not reached.

**Table 5:** Incidence and diagnosis of emergency department visits and hospital admission stratified by chemotherapy regimen.

Variables	Total (N = 10,634); (%)	TC (N = 2487)	ACT (N = 2981)	FEC-D (N = 5166)	*p* Value
Emergency department visits	3600 (33.9%)	988 (39.7%)	588 (19.7%)	2024 (39.2%)	<0.001
Most frequent diagnosis	Fever (720; 20%)	Fever (n = 206)	Fever (n = 130)	Neutropenia (n = 427)	
	Neutropenia (602; 16.7%)	Neutropenia (n = 164)	UTI (n = 34)	Fever (n = 384)	
	UTI (149; 4.14%)	Chest pain (n = 47)	Pneumonia (n = 25)	UTI (n = 77)	
	Chest pain (140; 3.9%)	Pneumonia (n = 40)	Sepsis (n = 21)	Chest pain (n = 77)	
	Acute URTI (n = 105)	UTI (n = 38)	Chest pain (n = 16)	Phlebitis (n = 65)	
	Pneumonia (n = 100)	Urticaria (n = 29)	Neutropenia (n = 11)	Acute URTI (n = 60)	
Hospitalization	1308 (12.3%)	344 (13.8%)	266 (8.9%)	698 (13.5%)	<0.001
Most frequent diagnosis	Neutropenia (n = 558)	Neutropenia (n = 156)	Fever (n = 23)	Neutropenia (n = 393)	
	Fever (n = 86)	Fever (n = 29)	Pneumonia (n = 14)	Fever (n = 43)	
	Pneumonia (n = 35)	Pneumonia (n = 11)	Neutropenia (n = 9)	N. V. (n = 16)	
Neutropenia MRD	855 (8%)	237 (9.5%)	26 (0.9%)	592 (11.5%)	<0.001
Febrile Neutropenia MRD	739 (6.9%)	198 (8.0%)	15 (0.5%)	526 (10.2%)	<0.001
Incidence of CHF	97 (0.9%)	25 (1.0%)	19 (0.6%)	53 (1.0%)	0.018
Mean ± S.D.	1083.81 ± 808.69	782.37 ± 702.04	868.5 ± 763.4	1364.11 ± 807.1	0.477
Median (Q1–Q3)	1039.5 (341–1608)	650 (150–1135)	489 (260–1271.5)	1476 (785–1973)	0.001

## Data Availability

The dataset from this study is held securely in coded form at ICES. Although legal data sharing agreements between ICES and data providers (e.g., health care organizations and government) prohibit ICES from making the dataset publicly available, access may be granted to those who meet the prespecified criteria for confidential access, and it is available at www.ices.on.ca/DAS (accessed on 19 December 2024) (email: das@ices.on.ca). The full dataset creation plan and underlying analytic code are available from the authors upon request, understanding that the computer programs may rely upon coding templates or macros that are unique to ICES and are, therefore, either inaccessible or may require modification.
